# P01.51. Light-emitting diode therapy reduces mechanical hypersensitivity induced by plantar incision model in mice via opioid mechanism

**DOI:** 10.1186/1472-6882-12-S1-P51

**Published:** 2012-06-12

**Authors:** F Cidral-Filho, L Mazzardo-Martins, D Martins, A OO Moré, A Soares dos Santos

**Affiliations:** 1 Federal University of Santa Catarina, Florianópolis, Brazil

## Purpose

Evaluate the antihypersensitivity effect of Light Emitting Diode Therapy (LEDT) in the plantar incision (PI) model in mice as well as investigate the possible involvement of the opioid system in this effect.

## Methods

The experiments were approved by the institution ethics committee under protocol n. 23080.006492/2011-61. Male Swiss mice were randomly divided in the following groups (n=8): naive, control (not treated), Off (LED device turned off), LEDT 1, 3, 5, 7 and 9 (treated with energy densities of 1 through 9 J/cm2). Control, Off and LEDT groups were submitted to a 5mm longitudinal PI (right hindpaw) under anesthesia (1-2% isoflurane). Mechanical hypersensitivity (MH) was assessed as withdrawal frequency percentage to 10 presentations of a 0.4g von Frey filament. Evaluations were conducted before and on day 1 through 5 after PI. LEDT (MOLIMEDpen® device; 950 nm wavelength, 80 mW/cm2 irradiance; 1 to 9 J/cm2 energy density) was applied directly to the skin of the incision site.

## Results

Results demonstrate that LEDT reduced MH in a dose-response manner with best results obtained with 9 J/cm2 (inhibition of 55±10% and effect lasting for 1 hour). Treatment with 1 J/cm2 and with LED device turned off did not reduce MH. Furthermore, LEDT as well as morphine (5 μg/site i.pl.) effects were blocked by intraperitoneal (i.p.), intraplantar (i.pl.) or intrathecal pre-administration of naloxone (1 mg/kg i.p.; 5 μg/site i.pl. or 5 μg/site i.t.) 20 minutes prior to LEDT or morphine treatment.

## Conclusion

LEDT reduced hypersensitivity induced by PI in mice via peripheral as well as central opioid mechanisms.

Sources of research support: UFSC, Capes.

